# Different Modulatory Effects of IL-17, IL-22, and IL-23 on Osteoblast Differentiation

**DOI:** 10.1155/2017/5950395

**Published:** 2017-07-27

**Authors:** Jing-Ru Zhang, Dan-Dan Pang, Qiang Tong, Xia Liu, Ding-Feng Su, Sheng-Ming Dai

**Affiliations:** ^1^Department of Rheumatology & Immunology, Changhai Hospital, Second Military Medical University, Shanghai, China; ^2^Department of Rheumatology & Immunology, The First Affiliated Hospital of Zhejiang Chinese Medical University, Zhejiang, China; ^3^Department of Rheumatology & Immunology, Shanghai Jiao Tong University Affiliated Sixth People's Hospital, Shanghai, China; ^4^Department of Pharmacology, School of Pharmacy, Second Military Medical University, Shanghai, China

## Abstract

**Objectives:**

To examine the expressions of IL-17, IL-22, and IL-23 receptors in four osteoblast models and the effects of IL-17, IL-22, and IL-23 on osteoblasts.

**Methods:**

Gene expression levels of receptors, alkaline phosphatase (ALP), osteocalcin (OCN), and Runt-related transcription factor 2 (Runx-2), were evaluated by RT-PCR and real-time RT-PCR. Proliferative responses and cell cycle analysis were detected by a CCK-8 assay and flow cytometry, respectively. ALP activity and ALP mass were detected by an ALP activity assay and ALP staining, respectively.

**Results:**

In primary osteoblasts, only the IL-17 receptor was expressed. In C2C12, MC3T3-E1, and Saos-2 cells, the genes of IL-17, IL-22, and IL-23 receptors were not detectable. None of IL-17, IL-22, and IL-23 had an obvious effect on the proliferation of primary osteoblasts, but IL-17 exhibited an inhibitory effect on the gene expression of ALP, OCN, and Runx-2. The ALP activity and ALP mass of primary osteoblasts were downregulated by IL-17 treatment in a dose-dependent manner, and IL-17 failed to inhibit BMP-2-induced phosphorylation of Smad.

**Conclusion:**

Primary osteoblasts constitutively express IL-17 receptors, but none of C2C12 cells, MC3T3-E1 cells, and Saos-2 cells express any receptors for IL-17, IL-22, and IL-23. IL-17 inhibits BMP-2-induced osteoblast differentiation via the BMP/Smad-independent pathway.

## 1. Introduction

Ankylosing spondylitis (AS) is a chronic inflammatory joint disease that chiefly affects the sacroiliac joints and the spine [1]. Radiographs reveal erosive changes at the corners of the vertebral bodies in the early stages of the disease and outgrowth of bony spurs known as syndesmophytes in the later stages [2]. When these syndesmophytes make the adjacent vertebral bodies fuse together, the spine appears as a single piece and is aptly described as a bamboo spine. The pathogenesis of syndesmophyte formation in AS remains unknown.

IL-23 is an immunomodulatory cytokine; the effects of which are mediated by downstream cytokines such as IL-17 and IL-22. Recently, accumulating data suggest that the IL-23/IL-17 axis plays a pivotal role in AS. One of the earliest discoveries that implicated IL-23 signaling in AS was an association with variants in the gene encoding one subunit of the IL-23 receptor (IL-23R) [3], and the association between the IL-23 receptor and AS was confirmed in subsequent studies of individuals of European descent [4] and Chinese population [5]. Subsequently, elevated IL-17 levels were found in the serum and synovial fluid of patients with active AS, undifferentiated spondyloarthropathy (SpA), and psoriatic arthritis (PsA) [6, 7]. Also, increased numbers of IL-23-responsive T cells (including Th17 cells, ROR*γ*t+ CD3+CD4−CD8− T cells, TCR*γδ*17 cells, KIR3DL2+CD4+ T cells, and iNKT17 cells) in the peripheral blood and synovial fluid of both patients with seronegative SpA and AS were found [8–13]. Moreover, higher expression of IL-23p19 mRNA in peripheral blood mononuclear cells (PBMC) of AS patients as well as enhanced production of IL-17 in the supernatants of PBMC stimulated by recombinant IL-23 was found [14]. These results have been confirmed by studies with animal models of SpA in which the number of Th17 cells and the levels of IL-23, IL-17, and IL-22 were systemically or locally elevated [13, 15–17]. Recently, the satisfactory efficacy of the anti-IL-17A monoclonal antibody secukinumab in treating patients with active AS also firmly implicates the involvement of IL-17 in the pathogenesis of AS [18]. The striking convergence of the evidences from clinical studies, genetic studies, animal models, and therapeutic trials strongly implicates that the IL-23/IL-17 axis plays a pivotal role in the pathogenesis of AS.

In addition, the possible role of the IL-23/IL-17 axis in the regulation of bone homeostasis is gradually being recognized. Some studies imply that IL-17 and IL-23 might regulate bone remodeling. For example, IL-17A promoted the production of RANKL by osteoblasts and synoviocytes and upregulated the expression of RANK on osteoclast precursors [19–22]. Moreover, it was recently reported that IL-23 stimulates the formation of functional osteoclasts in human PBMC cultures and the expression of RANK in mouse osteoclast progenitor cells [23, 24]. Langrish et al. [25] observed that lipopolysaccharide- (LPS-) driven osteoclast formation in vivo was reduced in the absence of IL-23. These data suggest the involvement of the IL-23/IL-17 axis in the regulation of bone homeostasis by inducing bone erosion in AS. Furthermore, new bone formation was found in the mice with overexpression of IL-23 [13]. However, the direct effects of the IL-23/IL-17 axis on osteoblasts or new bone formation of AS remain unknown.

In the present study, we aim to explore the possible effects of IL-17, IL-22, and IL-23 on osteoblasts. We first examined the expression levels of IL-17, IL-22, and IL-23 receptors in primary osteoblasts as well as in three representative osteoblast-like cell lines (C2C12, Saos-2, and MC3T3-E1). Furthermore, we detected the possible effects of IL-17, IL-22, and IL-23 on the proliferation and differentiation of osteoblasts. These studies will contribute to better understanding of the role of IL-17, IL-22, and IL-23 in the process of new bone formation. Based on these results, we look forward to making some advance in clarifying the role of the IL-17/IL-23 axis in the physiopathology of AS.

## 2. Materials and Methods

### 2.1. Reagents

Recombinant human bone morphogenetic protein-2 (BMP-2) was purchased from PeproTech Inc. (Minneapolis, MN, USA). Recombinant murine IL-17A, IL-22, and IL-23 were purchased from PeproTech Inc. (Rocky Hill, NJ, USA).

### 2.2. Cell Culture and Treatment

The mouse myoblast cell line C2C12, the murine preosteoblast cell line MC3T3-E1 subclone 14, and the human osteosarcoma cell line Saos-2 were purchased from Chinese Academy of Sciences (Shanghai, China). C2C12 cells were maintained in Dulbecco's modified Eagle's medium (DMEM) (Gibco, Grand Island, NY, USA) containing 15% fetal bovine serum (FBS) (Gibco, Grand Island, NY, USA) and antibiotics (100 U/ml of penicillin G and 100 *μ*g/ml of streptomycin) at 37°C in a humidified atmosphere of 5% CO_2_ in air. MC3T3-E1 cells were cultured in *α*-modified Eagle's medium (*α*-MEM) (Gibco, Grand Island, NY, USA) supplemented with 10% FBS and antibiotics (100 U/ml of penicillin G and 100 *μ*g/ml of streptomycin) at 37°C in a humidified atmosphere of 5% CO_2_ in air. Saos-2 cells were maintained in DMEM containing 10% FBS and antibiotics (100 U/ml of penicillin G and 100 *μ*g/ml of streptomycin) at 37°C in a humidified atmosphere of 5% CO_2_ in air. Primary rat calvarial osteoblasts were isolated from the calvaria of neonatal Sprague-Dawly rats (Animal Experimental Center of Second Military Medical University, Shanghai, China) by sequential collagenase digestion as described previously [26]. All animal experiments were undertaken in accordance with the National Institute of Health's “Guide for the Care and Use of Laboratory Animals”, with the approval of the Scientific Investigation Board of Second Military Medical University.

### 2.3. Induction of Osteoblast Differentiation

To induce osteoblast differentiation, MC3T3-E1, Saos-2, and primary calvarial osteoblasts were seeded (1 × 10^6^ cells/ml), and after 24 h incubation, rhBMP-2 at the final concentration of 300 ng/ml was added to the culture medium with 10% FBS. C2C12 cells were seeded (1 × 10^6^ cells/ml), and after 24 h incubation, the culture medium was replaced by DMEM supplemented with 5% FBS and 300 ng/ml rhBMP-2. All of the cells were maintained at 37°C in a humidified atmosphere of 5% CO_2_ in air, and the medium was replaced every 2 days.

### 2.4. Cell Proliferation Assay (CCK-8)

Cell proliferation was measured as previously described, using Cell Counting Kit-8 (CCK-8) according to the instruction of the manufacturer (Dojindo Laboratories, Kumamoto, Japan) [27]. Briefly, primary calvarial osteoblasts were seeded into 96-well plates (5 × 10^3^ cells/well). After 24 h incubation, the culture medium was refreshed and the cultivation was continued for 24–72 h in the presence of IL-17 (25 ng/ml), IL-22 (25 ng/ml), and IL-23 (25 ng/ml). After the incubation for 24–72 h, the supernatant was removed and 100 *μ*l CCK-8 solution was added to cells. After 4 h of incubation with CCK-8 at 37°C, absorbance was measured on an ELISA reader (Emax Science Corp., Sunnyvale, California, USA) at a wavelength of 450 nm.

### 2.5. Cell Cycle Analysis

Cell cycle profiles were analyzed as previously described [28]. Cells were seeded at 2 × 10^6^ cells per well in a 6-well plate and incubated for 24 h. Fresh medium containing IL-17 (25 ng/ml), IL-22 (25 ng/ml), or IL-23 (25 ng/ml) was added to the culture plate. After 24 h, the cells were collected, gently resuspended into a single cell suspension in phosphate-buffered saline (PBS), and fixed overnight with 70% ice-cold ethanol at 4°C. Cell pellets were harvested, then resuspended in 100 *μ*l RNase A solution, and incubated for 30 minutes at 37°C in a water bath. Then, 400 *μ*l propidium iodide (PI) staining solution was added, and the cells were incubated in the condition protected from light for another 30 min at 4°C. The percentage of cells in each of the S, G1, and G2/M phases of the cell cycle was determined by flow cytometry using the EPICS profile analyzer (Coulter Corp., Miami, FL, USA). The distribution in each phase of the cell cycle was determined using the ModFit LT 2.0 program, and the results were displayed as histograms.

### 2.6. Reverse Transcriptase Polymerase Chain Reaction (RT-PCR) and Quantitative Real-Time PCR

Total RNA was extracted from C2C12, MC3T3-E1, Saos-2, and primary calvarial osteoblasts by acid guanidine-phenol-chloroform extraction using the TRIzol Reagent (Invitrogen, California, USA). RNA was evaluated spectrophotometrically for quantity and purity. First-strand complementary DNA (cDNA) was synthesized from isolated RNA using PrimeScript™ RT Master Mix (Takara, Otsu, Japan) according to the manufacturer's protocol and used as templates for PCR. PCR amplification was performed using specific primers (Supplementary Table 1 available online at https://doi.org/10.1155/2017/5950395). The constitutively expressed gene encoding GAPDH was used as an internal control in RT-PCR to normalize the amounts of mRNA in each sample. The PCR products were analyzed by electrophoresis in 2% agarose gels stained with ethidium bromide, and bands were visualised and photographed under ultraviolet excitation. Quantitative real-time RT-PCR was performed on the 7500 Real-Time PCR System (Applied Biosystems, Foster City, CA, USA) using FastStart Universal SYBR Green Master (Roche, Germany). Data were adjusted by the levels of GAPDH expression in each sample.

### 2.7. Alkaline Phosphatase (ALP) Activity

After removing the culture medium, the cell layers were washed twice with PBS and lysed in 500 *μ*l of Tris-HCL (pH = 8.6). ALP activity in the cell lysate was assayed using an ALP testing kit (Nanjing Jiancheng Bioengineering Institute, Nanjing, China) following the instruction. The enzyme activity was expressed as micromoles of p-nitrophenol produced per min per mg of protein. The protein content was determined using the BCA Protein Assay kit (Beyotime, Shanghai, China) using BSA as the standard.

### 2.8. ALP Staining

Cells were washed twice with PBS and fixed with 4% paraformaldehyde at the room temperature for 10 min, followed by three additional ddH_2_O washes. Then, fixed cells were added with BCIP/NBT solution (Beyotime, Shanghai, China), following the instructions. Stained cell photos were taken using a digital camera.

### 2.9. Western Blotting Analysis

Proteins were extracted with an MPER protein extraction reagent (Pierce, Rockford, IL, USA) supplemented with the protease inhibitor mixture (Calbiochem, San Diego, CA, USA). The protein samples were subjected to SDS-PAGE using anti-phosphoSmad1/5/8 antibody and anti-GAPDH antibody (all from Cell Signaling, Danvers, MA, USA). The relative integrated density of each protein band was determined using an Odyssey Infrared Imaging System (LI-COR Bioscience, Lincoln, NE, USA).

### 2.10. Statistical Analysis

All experiments were repeated independently at least three times. Data are represented as mean ± SD. Student's *t*-test was used to determine the significance of differences between two groups, whereas analysis of variance (ANOVA) was used for multiple comparisons. Values of *p* < 0.05 were considered significant.

## 3. Results

### 3.1. Identification of Primary Calvarial Osteoblasts

**Figure 1:**
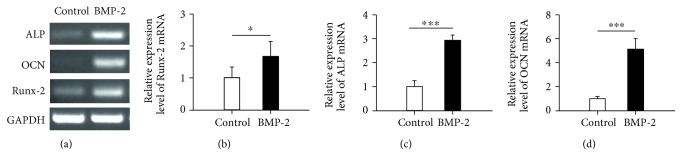
Identification of primary calvarial osteoblasts. The primary osteoblasts were isolated from calvaria of neonatal Sprague-Dawly rats. (a) The gene expression levels of alkaline phosphatase (ALP), osteocalcin (OCN), and Runx-2 were detected by RT-PCR after the cells were cultured in the absence or presence of recombinant human bone morphogenetic protein-2 (BMP-2) (300 ng/ml); GAPDH was used as a gel loading control. (b, c, d) The gene expression levels of ALP, OCN, and Runx-2 were analyzed by quantitative real-time RT-PCR. Compared with the cells without BMP-2 stimulation: ^∗^*p* < 0.05, ^∗∗∗^*p* < 0.001.

Initially, we investigated osteogenic characteristics of the primary cells isolated from neonatal rat calvaria. During the differentiating stage, osteoblasts can express and secrete many specific molecules, such as alkaline phosphatase (ALP), osteocalcin (OCN), and Runt-related transcription factor 2 (Runx2). ALP is considered the most abundant glycoprotein in the extracellular matrix, and it is expressed by osteoblasts at the early stage of differentiation [29]. OCN is secreted solely by osteoblasts at the late stage of differentiation [30], and Runx2 is the most important transcription factor regulating osteogenic differentiation and osteoblast activation [31]. In the present study, the results from RT-PCR analysis showed that the primary cells specifically expressed the gene of these osteogenic markers ALP, OCN, and Runx2. Furthermore, the gene expression levels of these three markers were remarkably elevated when the cells were stimulated by 300 ng/ml BMP-2 (Figure 1(a)). The consistent results were confirmed by quantitative analysis with real-time PCR (Figures 1(b), 1(c), and 1(d)). These results indicated that the primary cells possessed osteogenic properties. Thus, we used these primary osteoblastic cells in the following experiments.

### 3.2. mRNA Expression Levels of IL-17, IL-22, and IL-23 Receptors in Different Osteoblast Models

**Figure 2:**
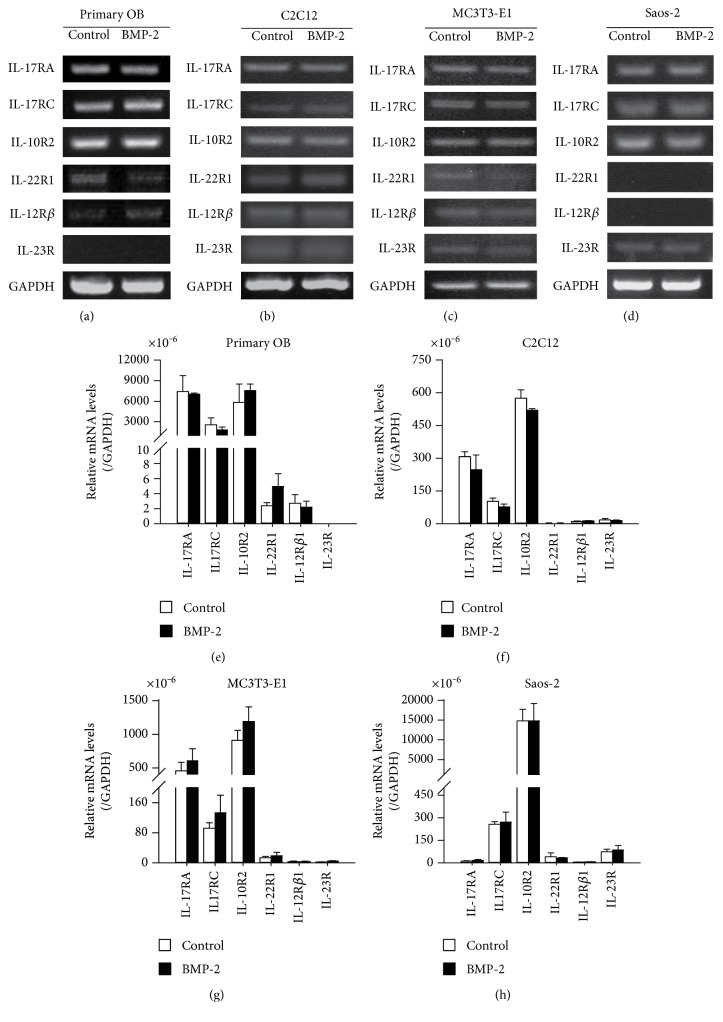
Gene expression levels of IL-17, IL-22, and IL-23 receptors in different osteoblast (OB) models. (a–d) The gene expression levels of IL-17, IL-22, and IL-23 receptors in primary osteoblasts, C2C12, MC3T3-E1, and Saos-2 were determined by RT-PCR analysis after the cells were cultured in the absence or presence of BMP-2 (300 ng/ml); GAPDH was used as a gel loading control. (e–h) The gene expression levels of IL-17, IL-22, and IL-23 receptors in primary osteoblasts, C2C12, MC3T3-E1, and Saos-2 were analyzed by quantitative real-time RT-PCR; the copies of the receptor mRNA was standardized with the copies of GAPDH mRNA.

It has been proved that IL-17A acts through a heterotrimeric receptor composed of two IL-17RA subunits and one IL-17RC subunit [32, 33]. Both the IL-22 receptor and IL-23 receptor are heterodimers. The IL-22 receptor consists of two subunits, IL-10R2 and IL-22R1 [34, 35]. The IL-23 receptor is composed of IL-12R*β*1 subunit and IL-23R subunit [36]. In rat primary osteoblasts, the RT-PCR analysis showed significant mRNA expression levels of IL-17RA, IL-17RC, and IL-10R2 subunits; marginal levels of IL-22R1 and IL-12R*β*1; and undetectable level of IL-23R (Figure 2(a)). Consistently, the results from quantitative real-time PCR confirmed that the mRNA copies of IL-17RA, IL-17RC, and IL-10R2 were above 1 × 10^−3^ standardized with the mRNA copies of GAPDH, while the mRNA copies of the other three subunits were all below 1 × 10^−3^ standardized with the mRNA copies of GAPDH (Figure 2(e)). Besides, there were no obvious changes in the expression levels of these receptor subunits in the presence or absence of BMP-2 stimulation. The above results demonstrated the presence of the IL-17 receptor and the absence of IL-22 and IL-23 receptors in rat primary osteoblasts.

In C2C12 cells, the mRNA of all the 6 interested subunits were mildly detected by semiquantitative RT-PCR, and quantitative real-time RT-PCR showed that the mRNA copies of all the 6 subunits were at the very low levels which were below 6 × 10^−4^ adjusted with the mRNA copies of GAPDH (Figures 2(b) and 2(f)). The similar results were observed in MC3T3-E1 cells in which the mRNA copies of IL-17RA, IL-17RC, IL-10R2, IL-22R1, IL-12R*β*1, and IL-23R were all below 1.5 × 10^−3^ adjusted with the mRNA copies of GAPDH (Figures 2(c) and 2(g)). In Saos-2 cells, only mRNA copies of IL-10R2 were above 1 × 10^−2^ standardized with the mRNA copies of GAPDH; the expressions of the other subunits were hardly detected (Figures 2(d) and 2(h)). Stimulation with BMP-2 failed to upregulate the expression levels of IL-17RA, IL-17RC, IL-10R2, IL-22R1, IL-12R*β*1, and IL-23R in C2C12 cells, MC3T3-E1 cells, or Saos-2 cells (Figure 2). These results demonstrated the absence of IL-17, IL-22, and IL-23 receptors in C2C12 cells, MC3T3-E1 cells, and Saos-2 cells.

### 3.3. Effects of IL-17, IL-22, and IL-23 on the Proliferation of Rat Primary Calvarial Osteoblasts

**Figure 3:**
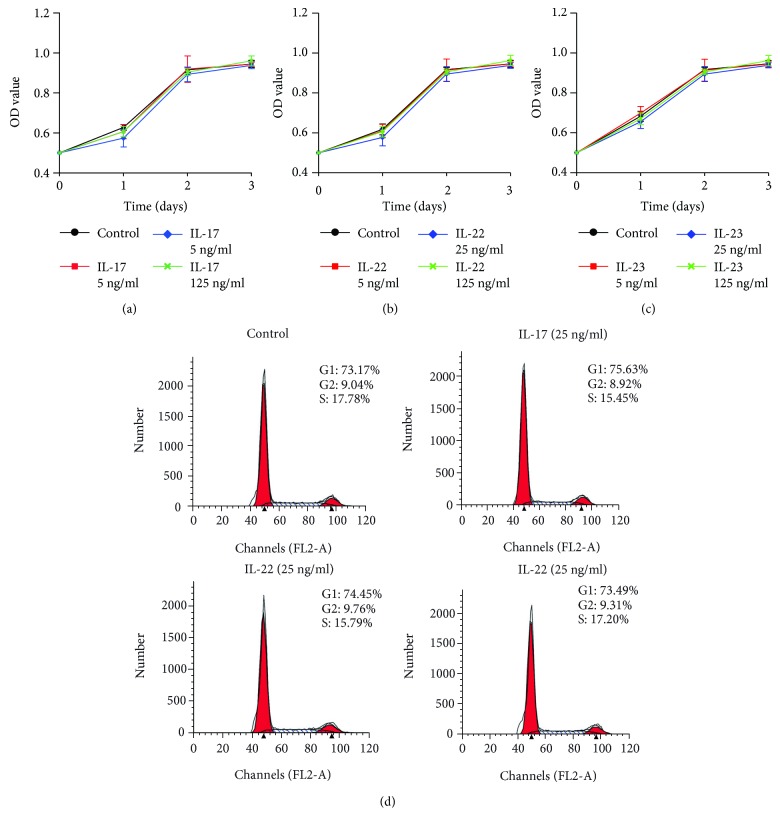
The effects of IL-17, IL-22, and IL-23 on the proliferation and cell cycle of the rat primary osteoblasts. (a–c) Proliferation of primary osteoblasts was tested by a CCK-8 assay after the cells were treated with various concentrations of IL-17, IL-22, and IL-23 (0, 5, 25, and 125 ng/ml) for different periods (24 h, 48 h, and 72 h). (d) Cell cycle analysis of primary osteoblasts were determined by flow cytometry after the cells were stimulated with or without IL-17 (25 ng/ml), IL-22 (25 ng/ml), and IL-23 (25 ng/ml) for 24 h.

The effects of IL-17, IL-22, and IL-23 on proliferation of rat primary calvarial osteoblasts were determined from cell growth kinetics with a CCK-8 assay measuring the metabolic activity of viable cells. As a result, all of IL-17 (5–125 ng/ml), IL-22 (5–125 ng/ml), or IL-23 (5–125 ng/ml) failed to induce the proliferation of osteoblasts (Figures 3(a), 3(b), and 3(c)). Besides, a cell cycle analysis was also conducted in primary osteoblasts treated with IL-17 (25 mg/ml), IL-22 (25 mg/ml), and IL-23 (25 mg/ml) for 24 h. Flow cytometric analysis showed that IL-17, IL-22, and IL-23 did not modulate the proportion of S phase cells compared with the control groups (Figure 3(d)).

### 3.4. Effects of IL-17, IL-22, and IL-23 on mRNA Expression of the Markers Associated with Osteogenic Differentiation in Primary Calvarial Osteoblasts

**Figure 4:**
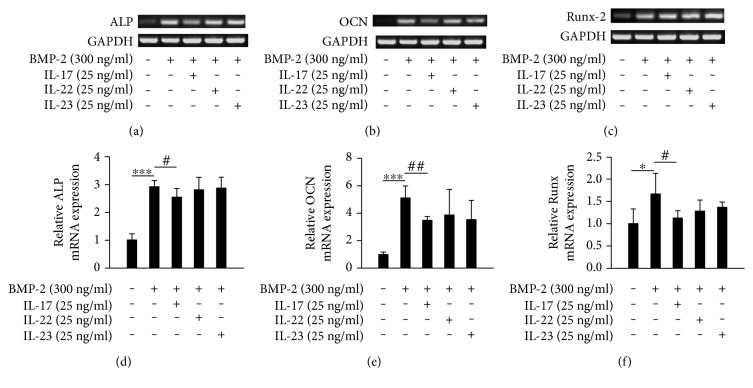
The effects of IL-17, IL-22, and IL-23 on mRNA expression of markers associated with osteogenic differentiation in primary calvarial osteoblasts. (a, b, c) Phenotypic markers of osteogenic differentiation (ALP, OCN, and Runx-2) were determined by RT-PCR in cells cultured with the differentiation inducer BMP-2 (300 ng/ml) in the presence or absence of IL-17 (25 mg/ml), IL-22 (25 mg/ml), or IL-23 (25 mg/ml) for 24 h. GAPDH was used as an internal control. (d, e, f) Phenotypic markers of osteogenic differentiation (ALP, OCN, and Runx-2) were analyzed by quantitative real-time RT-PCR. The copy number of ALP, OCN, and Runx-2 mRNA was standardized with the copy number of GAPDH mRNA. ^∗^*p* < 0.05, ^∗∗∗^*p* < 0.001 compared with untreated control; ^#^*p* < 0.05, ^##^*p* < 0.01 compared with the cells stimulated with BMP-2 alone.

The effects of IL-17, IL-22, and IL-23 on osteogenic differentiation were analyzed in primary calvarial osteoblasts. First, the mRNA expression levels of ALP, OCN, and Runx2 were analyzed in BMP-2-treated primary osteoblasts in the presence or absence of IL-17 (25 mg/ml), IL-22 (25 mg/ml), or IL-23 (25 mg/ml). Stimulation with BMP-2 significantly enhanced the mRNA expression levels of ALP, OCN, and Runx-2 in rat primary osteoblasts (Figures 4(a), 4(b), and 4(c)). IL-17 slightly inhibited the BMP-2-induced expression levels of ALP, OCN, and Runx-2 mRNA, while IL-22 and IL-23 failed to influence the BMP-2-induced mRNA expression levels of these differentiation markers (Figures 4(a), 4(b), and 4(c)). These results were further confirmed by quantitative real-time RT-PCR (Figures 4(d), 4(e), and 4(f)). These data demonstrated that IL-17 could inhibit osteogenic differentiation of rat osteoblasts, but IL-22 and IL-23 had a null effect on the differentiation.

### 3.5. Effects of IL-17, IL-22, and IL-23 on the ALP Activity of Primary Osteoblasts

**Figure 5:**
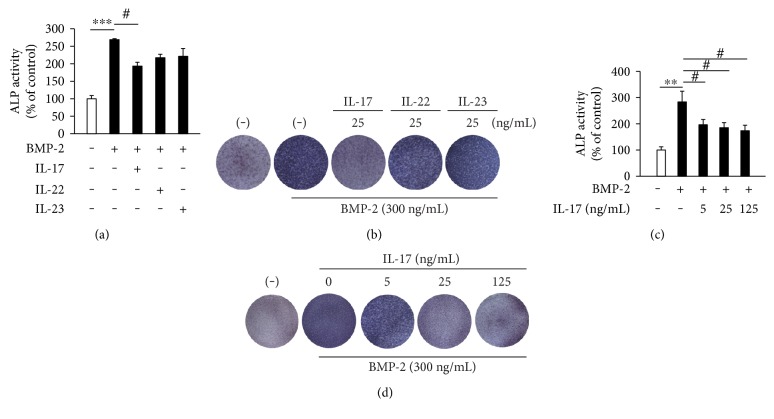
The effects of IL-17 on ALP activity and ALP mass of primary osteoblasts. (a) ALP activity in primary osteoblasts cultured with BMP-2 (300 ng/ml) in the presence of IL-17 (25 ng/ml), IL-22 (25 ng/ml), or IL-23 (25 ng/ml) for 7 days. ^∗∗∗^*p* < 0.001 compared with untreated control; ^#^*p* < 0.05 compared with the cells stimulated with BMP-2 alone. (b) Staining for ALP in primary osteoblasts cultured with BMP-2 (300 ng/ml) in the presence of IL-17 (25 ng/ml), IL-22 (25 ng/ml), or IL-23 (25 ng/ml) for 7 days. (c) ALP activity in primary osteoblasts cultured with BMP-2 (300 ng/ml) in the presence of different concentrations of IL-17 (0, 5, 25, and 125 ng/ml) for 7 days. ^∗∗^*p* < 0.01 compared with untreated control; ^#^*p* < 0.05 compared with the cells stimulated with BMP-2 alone. (d) Staining for ALP in primary osteoblasts cultured with BMP-2 (300 ng/ml) in the presence of different concentrations of IL-17 (0, 5, 25, and 125 ng/ml) for 7 days.

To further validate the effects of IL-17, IL-22, and IL-23 on the osteoblastic differentiation in primary calvarial osteoblasts, ALP staining and an ALP activity assay were carried out. The primary osteoblasts were induced to be differentiated by BMP-2 (300 ng/ml) and were further treated with or without IL-17 (25 ng/ml), IL-22 (25 ng/ml), and IL-23 (25 ng/ml) for 7 days. With the induction by BMP-2, the ALP activity in primary osteoblasts was remarkably elevated, which was reduced by IL-17, but not by IL-22 or IL-23 (Figure 5(a)). The inhibitory effect of IL-17 (5–125 ng/ml) on ALP activity was dose-dependent (Figure 5(c)). In the ALP staining test, it was confirmed that the BMP-2-induced ALP activity was inhibited by IL-17 in a dose-dependent manner but not affected by IL-22 or IL-23 (Figures 5(b) and 5(d)).

### 3.6. IL-17 Inhibits BMP-2-Induced Osteoblastic Differentiation via the BMP/Smad-Independent Pathway

**Figure 6:**
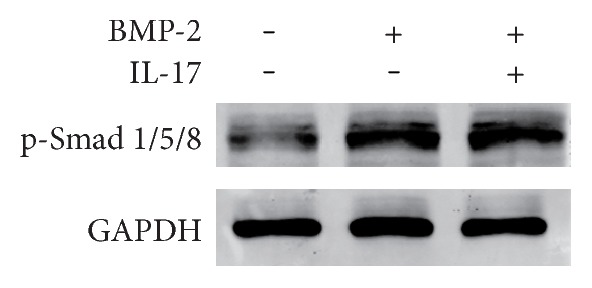
Effect of IL-17 on BMP-2-induced phosphorylation of Smad1/5/8. The rat primary osteoblasts were treated with BMP-2 (300 ng/ml) in the presence or absence of IL-17 (25 ng/ml) for 48 h. The intracellular level of phosphorylated Smad1/5/8 (p-Smad1/5/8) was analyzed by Western blotting.

BMP-2 is a potent osteoblastic differentiation inducer which signals though Smads. To determine whether IL-17 exerts an inhibitory effect on BMP-2-induced osteoblastic differentiation through crosstalk with the canonical BMP/Smad signaling, rat primary osteoblasts were treated with BMP-2 in the presence or absence of IL-17. The changes in phosphorylated Smad1/5/8 were analyzed by Western blotting. When the cells were stimulated with BMP-2 alone, Smad1/5/8 phosphorylation significantly increased. Treatment with IL-17 did not affect the BMP-2-induced phosphorylation of Smad1/5/8 (Figure 6).

## 4. Discussion

For the first time, it was shown that rat primary osteoblasts constitutively expressed both subunits of IL-17 receptors, but BMP-2, a potent inducer of osteoblast differentiation, did not modulate the expression level of IL-17 receptors. Moreover, IL-17 significantly inhibited the differentiation of osteoblasts induced by BMP-2. The rat osteoblasts did not express the intact receptors for IL-22 or IL-23, and neither IL-22 nor IL-23 modulated the differentiation of the osteoblasts. The most commonly used osteoblast-like cell lines, C2C12 cells, MC3T3-E1 cells, and Saos-2 cells, were lack of the expression of IL-17, IL-22, and IL-23 receptors.

Chronic inflammation and pathologic osteogenesis are two major characteristics of AS. Previous studies have revealed that a series of immune disorders exist in AS patients [8, 37]. However, the mechanisms of pathologic osteogenesis and the relationship between inflammation and pathologic osteogenesis have not yet been fully elucidated. Some researchers have suggested that the development of chronic inflammation and osteogenesis may not be synchronized in AS, indicating that pathologic osteogenesis is a relatively independent process with little relation to chronic inflammation [38–40]. Conversely, results of other studies consider chronic inflammation, which predominates in AS, to be the main cause of pathologic osteogenesis [41, 42]. Furthermore, several previous studies have shown that the IL-23/IL-17 axis exerts potent effects on bone remodeling which indicates the relationship between inflammatory factors and bone formation. In a murine model with overexpression of IL-23, Sherlock et al. [13] demonstrated the pivotal role for the cytokines IL-23 and IL-22 in the development of enthesitis and bone formation, respectively. So we tried to study the direct effects of IL-17, IL-22, and IL-23.

At the first step, we analyzed the expression levels of IL-17, IL-22, and IL-23 receptors in rat primary osteoblasts and the most commonly used osteoblast-like cell lines.

The murine multidifferentiating potential cell line C2C12, the cell line Saos-2 derived from human osteosarcoma, and the murine preosteoblast cell line MC3T3-E1 all have been demonstrated to have significant osteogenic characteristics [43–45]. It is well known that the primary osteoblast is an optimal choice for bone researches in vitro, but it has some disadvantages in the limitation and inconvenience of acquisition of cells. On the other hand, various osteoblast-like cell lines derived from different sources are with multiple advantages, such as the ready availability of large numbers of cells, the homogeneity of the cell cultures, and the expected invariability of the phenotype. Unfortunately, none of the selected osteoblast cell lines express the both subunits of the receptors for IL-17, IL-22, or IL-23. Previous studies have proved that the gene expression and the levels of osteogenic phenotypes vary greatly when the primary osteoblasts and osteoblast-like cells are at the different differentiation stages [29, 40, 41]. Thus, we further detected the gene expression levels of the above receptors in these cell lines during differentiation induced by BMP-2. However, BMP-2 failed to upregulate the expression levels of the above receptors in these cells. In the osteoblast cell line CRL-12424, IL-22 was shown to upregulate mRNA expression of factors that promote bone formation, including Wnt10b, BMP4, and ALP [13]. However, we failed to purchase CRL-12424 cells, and the reason for inconsistent results of IL-22 in the osteoblast cell lines remains unknown. So we have to use the rat primary osteoblasts in the following experiments.

According to the results from semiquantitative and quantitative RT-PCR analysis, IL-17 receptors (IL-17RA and IL-17RC) were clearly shown while IL-22 receptors and IL-23 receptors were absent in the rat primary osteoblasts. These results are consistent with those of previous studies which demonstrated the presence of IL-17 receptors in rat primary osteoblasts [46] and the absence of IL-23 receptors in mouse primary osteoblasts [47]. Moreover, no matter whether the cells were stimulated with BMP-2 or not, the mRNA expression levels of IL-17, IL-22, and IL-23 receptors remained the same.

At the next step, we studied the direct effects of IL-17, IL-22, and IL-23 on osteoblasts in vitro. Consistent with the deficit of IL-22 and IL-23 receptors in rat primary osteoblasts, both IL-22 and IL-23 failed to show any effects on the proliferation and differentiation. Although IL-17 did not modulate the proliferation of rat osteoblasts, IL-17 inhibited the BMP-2-induced differentiation of rat primary osteoblasts in a dose-dependent manner, which further supported the expression of the IL-17 receptor by rat osteoblasts. These results are consistent with the report by Kim et al. [46], in which IL-17 inhibited osteoblast differentiation and bone regeneration in rats. On the contrary, Huang et al. [48] found that IL-17 stimulated the proliferation of human mesenchymal stem cells (MSCs) and induced MSCs to differentiate into osteoblasts.

This kind of paradoxical effects on bone homeostasis is also found in TNF-*α*. TNF-*α* which has been proved to involve in the pathogenesis of AS classically is supposed to play a pivotal role in the regulation of bone homeostasis by stimulating osteoclastogenesis and inhibiting osteoblast function [49–51]. However, opposite findings suggest that TNF-*α* may also induce osteogenic differentiation [52–54]. This paradoxical effect of TNF-*α* in inhibiting or facilitating osteoblastogenesis lies in the differentiation stage of the responding cells [55]. Similarly, the inconsistent effects of IL-17 on osteogenesis may also be influenced by the differentiation state of the cell type, as MSCs are pluripotent progenitor cells that can differentiate into a variety of cell types.

Overall, IL-17, IL-22, and IL-23 did not show direct promoting effect on rat primary osteoblasts in vitro, but these data did not rule out any indirect effects of these inflammatory factors to facilitate osteoblast differentiation in vivo, which are awaiting further studies to clarify. The interplay between inflammatory factors and new bone formation in AS is still challenging.

BMP-2 modulates osteoblast differentiation through the canonical BMP/Smad pathway and noncanonical BMP pathways [56–58]. In the canonical BMP/Smad pathway, the activated BMP receptors subsequently propagate the BMP signals by phosphorylating BMP-specific Smad1/5/8. Then, the phosphorylated Smad1/5/8 binds to Smad4, and the complex is transported to the nucleus to activate the osteogenic-specific transcription factor Runx2. Runx2 has an essential role in osteoblast differentiation of stem cells and directly stimulates transcription of its important downstream target genes, including OCN, ALP, type I collagen (COL1A1), and osteopontin (OPN). In the present study, IL-17 failed to inhibit the phosphorylation of Smad1/5/8, which was induced by BMP-2. This finding suggests that the inhibitory effect of IL-17 on BMP-2-induced osteoblast differentiation is independent of the BMP/Smad signaling pathway, which is also supported by other studies. For example, TNF-*α* suppressed the expression of Runx2 and the transcription of osteogenic genes by interfering with the DNA binding of Smad proteins instead of inhibiting phosphorylation of Smad1/5/8 or nuclear translocation of the Smad1/Smad4 complex [59]. Huang et al. [60] found that TNF-*α*/IL-1*β* regulated BMP-2-induced osteoblastic differentiation by activating MAPK signaling pathways instead of the BMP-2/Smad signaling pathway. Therefore, these data suggest the existence of alternative pathways besides BMP/Smad signaling to regulate BMP-2-induced osteoblast differentiation.

In conclusion, rat primary osteoblasts constitutively express IL-17 receptors but lack IL-22 and IL-23 receptors. None of C2C12 cells, MC3T3-E1 cells, and Saos-2 cells express receptors for IL-17, IL-22, and IL-23. IL-17 inhibits BMP-2-induced osteoblast differentiation via the BMP/Smad-independent pathway.

## Supplementary Material

Table 1 Sequences of PCR primers, length of PCR product, optimal annealing temperature, and sequences accession number.
