# Supplementary material for: Different Modulatory Effects of IL-17, IL-22, and IL-23 on Osteoblast Differentiation
Source: Mediators Inflamm. 2017 Jul 27;2017:5950395. doi: 10.1155/2017/5950395 (PMC5555000; doi:10.1155/2017/5950395)
Supplement: Supplementary file 1 — Table 1 Sequences of PCR primers, length of PCR product, optimal annealing temperature, and sequences accession number. [file 5950395.f1.docx]

**Table 1** Sequences of PCR primers, length of PCR product, optimal annealing temperature, and sequences accession number.

| Primer | species | Accession  number | Sequence  (5’-3’) | Product  (bp) | Tm(°C) |
| --- | --- | --- | --- | --- | --- |
| IL-17RA | mouse | NM_008359.2\| | Fw: CCGACAGAAGCAGGAGATG | 100 | 59.5 |
|  |  |  | Rv: CTCAGCCCAACCCAAGATAG |  |  |
|  | rat | NM_001107883.2 | Fw: TTTACCCTCGAAACCTGACG | 120 | 60 |
|  |  |  | Rv: CTGTCTGCAAGGTCCACTCA |  |  |
|  | human | NM_001289905.1 | Fw: AGAACCAATTCCGGGGCCT | 169 | 60.8 |
|  |  |  | Rv: CTGGGCGAATTTCAGGACCA |  |  |
| IL-17RC | mouse | NM_134159.4 | Fw: CCGTGGGTTCTGCGGTATTT | 131 | 59.9 |
|  |  |  | Rv: GACATTGTCACCATCAGGCAG |  |  |
|  | rat | NM_001170565.1 | Fw: GGTGGTGCTCTCCTTTCAGG | 169 | 60 |
|  |  |  | Rv: CCTGGGTTTGGTGTAGGACC |  |  |
|  | human | NM_001203263.1 | Fw: TGCAGCTATGGGACGATGAC | 153 | 59.5 |
|  |  |  | Rv: CCGCTTTCGCGTGATCC |  |  |
| IL-10R2 | mouse | NM_008349.5 | Fw: CTTCTGGTGCCAGCTCTAGG | 100 | 59.9 |
|  |  |  | Rv: GGAAAGCAGGTACCTCCCAC |  |  |
|  | rat | NM_001107111.1 | Fw: ACGGACAACCAGTGATGAAACA | 171 | 60 |
|  |  |  | Rv: TTGAGGTGCTGTGGAAGAGAC |  |  |
|  | human | NM_000628.4 | Fw: GTGAGCCTGTCTGTGAGCAA | 143 | 60 |
|  |  |  | Rv: TGTCTTCTTGTAAACGCACCA |  |  |
| IL-22R1 | mouse | NM_178257.2 | Fw: GCACCTCTGACACCGTCTAC | 231 | 59.9 |
|  |  |  | Rv: GCGGTTTGATGGTAGTGTGC |  |  |
|  | rat | NM_001191869.1 | Fw: ATCTTGACGTGGGATGCTGG | 122 | 60 |
|  |  |  | Rv: AACTTCTGGGTGATCCGCTG |  |  |
|  | human | NM_021258.3 | Fw: CTCTGCAGCACACTACCCTC | 188 | 60 |
|  |  |  | Rv: CATTTGGTAGGTGCGGTTGAC |  |  |
| IL-12Rβ1 | mouse | NM_008353.2 | Fw: TTCAGCCCTGCAGAAGTTCC | 119 | 61.6 |
|  |  |  | Rv: GCCTGAGGCGCCTAGCTG |  |  |
|  | rat | NM_001170604.1 | Fw: TTACCGCATCACTGTCTTCG | 104 | 60 |
|  |  |  | Rv: TTCCGACCCTTGAGACGTTA |  |  |
|  | human | NM_001290024.1 | Fw: TTCAGCATCGAAGTGCAGGT | 104 | 60 |
|  |  |  | Rv: TTCAGGCCAAGGTAGCCAAG |  |  |
| IL-23R | mouse | NM_144548.1 | Fw: GGTGTCACGGAGGAATCACA | 232 | 59.9 |
|  |  |  | Rv: GCATGAGGTTCCGAAAAGCC |  |  |
|  | rat | XM_003753910.3 | Fw: GGCATGGAGAACTCACAACA | 110 | 59.8 |
|  |  |  | Rv: ATGGTCTTGGGCACATTAGC |  |  |
|  | human | XM_005270516.2 | Fw: CCCTGGAGACAAGAGACTACC | 133 | 57.9 |
|  |  |  | Rv: TTATTGCTGAGATGGCTTCC |  |  |
| ALP | mouse | XM_006538499.2 | Fw: AAGCTGGGAAGAACACTCCA | 59.5 | 100 |
|  |  |  | Rv: CAAACAGGAGAGCCACTTCA |  |  |
|  | rat | NM_053650.1 | Fw: GCAGCACTCCTTCCGGTATT | 156 | 60 |
|  |  |  | Rv: GTACAATCTTCACGCCCGGA |  |  |
|  | human | XM_005245820.2 | Fw: CACCCACGTCGATTGCATCT | 211 | 60.8 |
|  |  |  | Rv: TAGCCACGTTGGTGTTGAGC |  |  |
| OCN | mouse | NM_008318.3 | Fw: GCCCTTAGCCTTCCATAGTG | 143 | 59.5 |
|  |  |  | Rv: CGGGATACACACACCCTCTT |  |  |
|  | rat | NM_013414.1 | Fw: GGTGGTGAATAGACTCCGGC | 117 | 60 |
|  |  |  | Rv: AGCTCGTCACAATTGGGGTT |  |  |
|  | human | NM_199173.5 | Fw: AGCTCCCAACCACAATATCCT | 148 | 59 |
|  |  |  | Rv: TTATACCCTCTGGGCTGTGC |  |  |
| Runx | mouse | XM_006523548.2 | Fw: ACTCTGGGTCTGGGAGGAAG | 104 | 60 |
|  |  |  | Rv: GACTCTGTAAGCGGGTCTGC |  |  |
|  | rat | NM_001278484.2 | Fw: CCACCACTCACTACCACACG | 119 | 60 |
|  |  |  | Rv: GGACGCTGACGAAGTACCAT |  |  |
|  | human | XM_011514966.1 | Fw: GGAGTGGACGAGGCAAGAGTTT | 133 | 62.5 |
|  |  |  | Rv: AGCTTCTGTCTGTGCCTTCTGG |  |  |
| GAPDH |  | NM_002046 | Fw: GAAGGTGAAGGTCGGAGTC | 226 | 60 |
|  |  |  | Rv: GAAGATGGTGATGGGATTTC |  |  |
